# Combined Metabolite and Transcriptomic Profiling Unveil a Potential Gene Network Involved in the Triterpenoid Metabolism of *Rose roxburghii*

**DOI:** 10.3390/ijms25105517

**Published:** 2024-05-18

**Authors:** Liangqun Li, Mei Peng, Yanfang Yan, Tingfei Deng, Qiancheng Liang, Xian Tao, Haodong Li, Juan Yang, Guandi He, Sanwei Yang, Xiaojun Pu, Xiaosheng Yang

**Affiliations:** 1 State Key Laboratory of Functions and Applications of Medicinal Plants, Guizhou Medical University, Guiyang 550014, China; liliangqun2010@163.com (L.L.); yanfangyan1926@sina.com (Y.Y.); dengtingfie@sina.com (T.D.); 18308579886@163.com (Q.L.); 18942578550@163.com (H.L.); yangxz2002@126.com (J.Y.); 2 Natural Products Research Center of Guizhou Province, Guiyang 550014, China; 3 College of Agriculture, Guizhou University, Guiyang 550025, China; gdhe@gzu.edu.cn (G.H.); swyang@gzu.edu.cn (S.Y.); 4 Faculty of Life Science and Technology, Kunming University of Science and Technology, Kunming 650031, China

**Keywords:** *Rose roxburghii*, triterpenoids, transcriptome profiling, biosynthesis, high-performance liquid chromatography HPLC

## Abstract

*Rose roxburghii*, a horticulturally significant species within the *Rosa* genus of the Rosaceae family, is renowned for its abundance of secondary metabolites and ascorbate, earning it the title ‘king of vitamin C’. Despite this recognition, the mechanisms underlying the biosynthesis and regulation of triterpenoid compounds in *R. roxburghii* remain largely unresolved. In this study, we conducted high-performance liquid chromatography profiling across various organs of *R. roxburghii*, including fruit, root, stem, and leaves, revealing distinct distributions of triterpenoid compounds among different plant parts. Notably, the fruit exhibited the highest total triterpenoid content, followed by root and stem, with leaf containing the lowest levels, with leaf containing the lowest levels. Transcriptomic analysis unveiled preferential expression of members from the cytochrome P450 (*CYP*) and glycosyltransferase (*UGT*) families, likely contributing to the higher accumulation of both ascorbate and triterpenoid compounds in the fruits of *R. roxburghii* compared to other tissues of *R. roxburghii*. Transcriptomic analysis unveiled a potential gene network implicated in the biosynthesis of both ascorbate and triterpenoid compounds in *R. roxburghii*. These findings not only deepen our understanding of the metabolic pathways in this species but also have implications for the design of functional foods enriched with ascorbate and triterpenoids in *R. roxburghii*.

## 1. Introduction

*Rosa roxburghii* Tratt., belonging to the *Rosa* genus of the Rosaceae family, is a species of profound horticultural and medicinal importance, particularly in Guizhou Province, situated in southwestern China [1,2]. The region’s food and pharmaceutical sectors greatly benefit from this species owing to the rich assortment of bioactive compounds found in its fruits, including vitamin C, amino acids, flavonoids, and terpenoids [1,2,3]. These compounds exhibit notable pharmacological effects, encompassing antibacterial, anti-aging, anti-apoptotic, antioxidant, and anti-atherosclerotic properties [4,5,6]. With advancing research, the potential health advantages of *R. roxburghii* have attracted widespread attention.Currently, Guangzhou Pharmaceutical Group Co., Ltd. has successfully developed various products from the fruit of *R. roxburghii*, including juice, vinegar, jam, and cakes, etc., which generate considerable economic benefits with sales exceeding RMB 100 million [6]. Furthermore, in recent years, some high-quality and high-yielding varieties of *R. roxburghii*, such as “Guinong No. 5”, “Guinong No. 57”, and “Guinong No. 95”, have been successively bred. Among them, Guinong No. 5 is favored for its characteristics of heavier single fruit and higher content of *R. roxburghii* juice [6]. Although many *R. roxburghii* fruits contain triterpenes, the current understanding of the biosynthetic pathways of triterpenes and the crucial regulatory genes involved in fruits of *R. roxburghii* remains very limited. 

Triterpenoids, among the myriad bioactive components, have garnered significant interest due to their distinctive structures and diverse biological activities [7,8,9]. In plants, sesquiterpenoids and triterpenoids are synthesized from precursors isopentenyl diphosphate (IPP) and dimethylallyl diphosphate (DMAPP), which are derived from the mevalonate (MVA) pathway in the cytoplasm and the methylerythritol phosphate (MEP) pathway in the plastid [10,11]. IPP is then condensed with DMAPP to generate geranyl pyrophosphate (GPP) through the catalysis of geranyl pyrophosphate synthase (GPS) and/or farnesyl pyrophosphate (FPP) through the catalysis of farnesyl diphosphate synthase (FPS) [10]. Subsequently, the two FPP molecules are condensed by squalene synthase (SS) to generate squalene, which is epoxidized to 2,3-oxidosqualene through the catalysis of squalene epoxidase (SE). Afterward, 2,3-oxidosqualene undergoes cyclization catalyzed by oxidosqualene cyclase (OSC) to form a variety of tetracyclic and pentacyclic triterpenoid skeletons. These skeletons can be modified by the cytochrome P450 (*CYP*) family and the glycosyltransferase (UGT) family to generate various pentacyclic triterpenoids [11,12]. 

The biosynthesis of triterpenoids in plants is associated with several biological processes, such as growth and development, defense responses, and interactions with environmental stimuli [13,14,15,16,17,18]. Studies have revealed that triterpenoids sourced from *R. roxburghii* possess multifaceted functionalities, ranging from treating depression and improving sleep, to managing enteritis, indicating their potential in various health domains [1,19,20]. Pentacyclic triterpenoids emerge as the primary constituents in *R. roxburghii* [19]. Given the pivotal role of the *R. roxburghii* industry in Guizhou Province, a comprehensive understanding of pentacyclic triterpenoid biosynthesis and regulation in *R. roxburghii* is important for further industry development, particularly in improving fruit quality. In this study, we chose the good-quality variety “Guinong No. 5” as the material to be examined, and conducted metabolite and transcriptomic profiling to elucidate the distribution of triterpenoids and identify key genes and regulatory factors potentially associated with triterpenoid biosynthesis in *R. roxburghii*. Our findings not only provide scientific evidence for the good quality of Guinong No. 5, but also have implications for designing fruits with enhanced accumulation of triterpenoids and ascorbate.

## 2. Results

### 2.1. Triterpenoid Compound Accumulation in R. roxburghii

**Figure 1:**
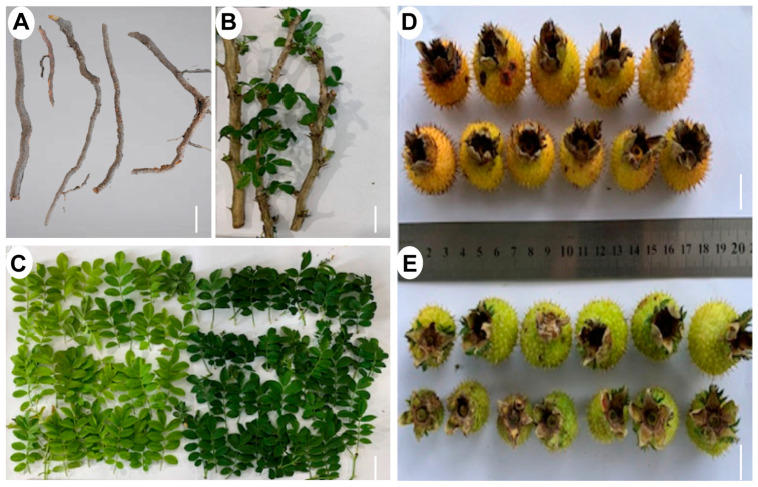
Typical images of *R. roxburghii* from different tissues: (**A**) Root. (**B**) Stem. (**C**) Young leaves and old leaves. (**D**) Matured fruit. (**E**) Young fruits. Bar = 2 mm.

To investigate the main triterpenoid compound profiles, we collected various organs of *R. roxburghii*, including roots, stems, leaves, and fruits (Figure 1). 

**Figure 2:**
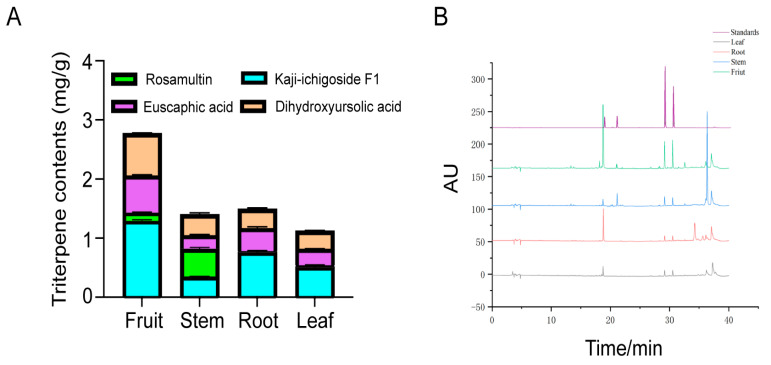
(**A**) Triterpene contents in *R. roxburghii* of different tissues by HPLC (Mean ± standard deviation, n = 3). Means showing significant differences (*p* < 0.05) are labeled with different letters based on one-way ANOVA with Duncan’s multiple range test. (**B**) Chromatograms of *R. roxburghii* samples from different tissues. The purple line represents four standards: green line for fruit samples, blue line for stem samples, red line for root samples, and black line for leaf samples. I. Kaji-ichigoside F1; II. Rosamultin; III. Euscaphic acid; IV. 2α,19α-Dihydroxyursolic acid.

The distribution of triterpenoid compounds across various organs of *R. roxburghii* was investigated through HPLC analysis. Four pentacyclic triterpenoid components, namely kaji-ichigoside F1, rosamultin, euscaphic acid, and 2α,19α-dihydroxyursolic acid, were quantified in the root, stem, leaves, and fruit. Results revealed distinct accumulation patterns among different plant parts (Figure 2A).

Kaji-ichigoside F1, Euscaphic acid and 2α,19α-dihydroxyursolic acid exhibited the highest concentration in the fruits (Table 1 and Figure 2A), indicating fruit tissues as the primary reservoir for these compounds. In contrast, Rosamultin was predominantly found in stems, with a content of 0.48 ± 0.02 mg/g, followed by fruits with a content of 0.14 ± 0.01 mg/g (Table 1 and Figure 2A). In addition to the fruit, the levels of kaji-ichigoside F1 and euscaphic acid are also relatively high in the roots of *R. roxburghii*, indicating that roots are also accumulation sites for these triterpenoids (Table 1 and Figure 2A). The total triterpenoid content varied across different organs, with the highest accumulation observed in fruits, followed by roots and stems. Leaves exhibited the lowest total triterpenoid content among the analyzed organs (Figure 2A).

### 2.2. Comparative Analysis of Differentially Expressed Genes 

**Figure 3:**
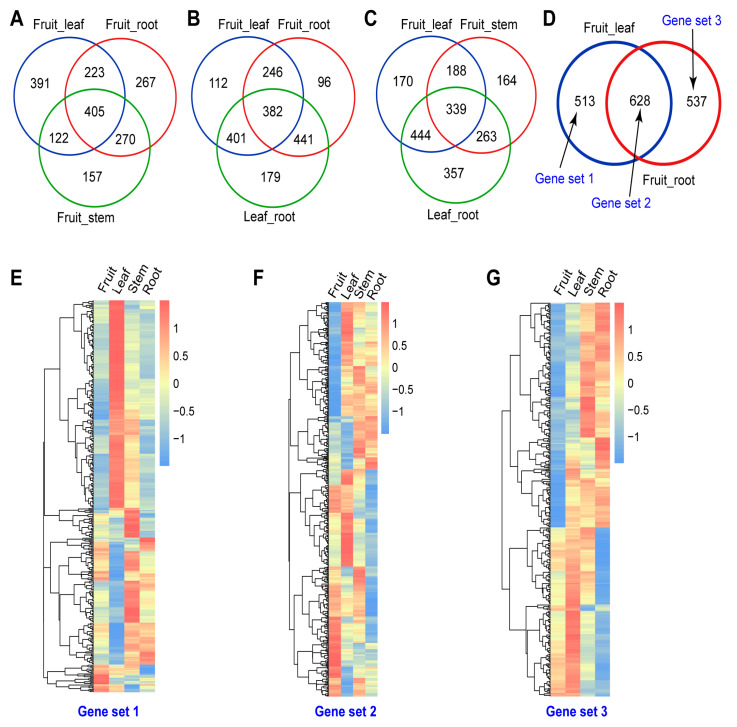
Venn diagram showing the differentially expressed genes in various organs of *R. roxburghii*. (**A**) Venn diagram showing the differentially expressed genes between fruit and leaves, fruit and roots, and fruit and stems. (**B**) Venn diagram showing the differentially expressed genes between fruit and leaves, fruit and roots, and leaves and roots. (**C**) Venn diagram showing the differentially expressed genes between fruit and leaves, fruit and stems, and leaves and roots. (**D**) Venn diagram showing the differentially expressed genes between fruit and leaves, and fruit and roots. (**E**) Heatmap displaying the expression pattern of preferentially expressed genes in gene set 1, (**D**). (**F**) Heatmap displaying the expression pattern of fruit-preferentially expressed genes in gene set 2, (**D**). (**G**) Heatmap displaying the expression pattern of root-preferentially expressed genes in gene set 3, (**D**).

To identify genes that were preferentially expressed in different organs including roots, stems, leaves and fruits, the differentially expressed genes (DEGs) of different organs were analyzed in the RNA-seq datasets. As the fruit of *R. roxburghii* is the main edible part with higher economic value, we used it as a control to analyze differential gene expression by comparing it with other organs. We utilized log2(FoldChange) ≥ 1 and *p* < 0.05 as threshold to determine DEGs. We identified 1141, 1165, and 954 DEGs in leaves, roots, and stems, respectively, when fruits were used as the control. Additionally, 1403 DEGs were identified in roots when leaves were used as the control (Figure 3A–C, and Table S1). 

Furthermore, a Venn diagram revealed that 513 genes were preferentially expressed in the leaves (gene set 1), 628 were shared between the DEG datasets of fruits and leaves and fruits and roots (gene set 2), and 537 were expressed in the roots (gene set 3) (Figure 3D). Most of the genes in these three gene sets exhibited preferential expression patterns (Figure 3E–G).

### 2.3. Genes Involved in Vitamin C, Sesquiterpenoid and Triterpenoid Biosynthesis Were Significantly Up-Regulated in Fruit of R. roxburghii

**Figure 4:**
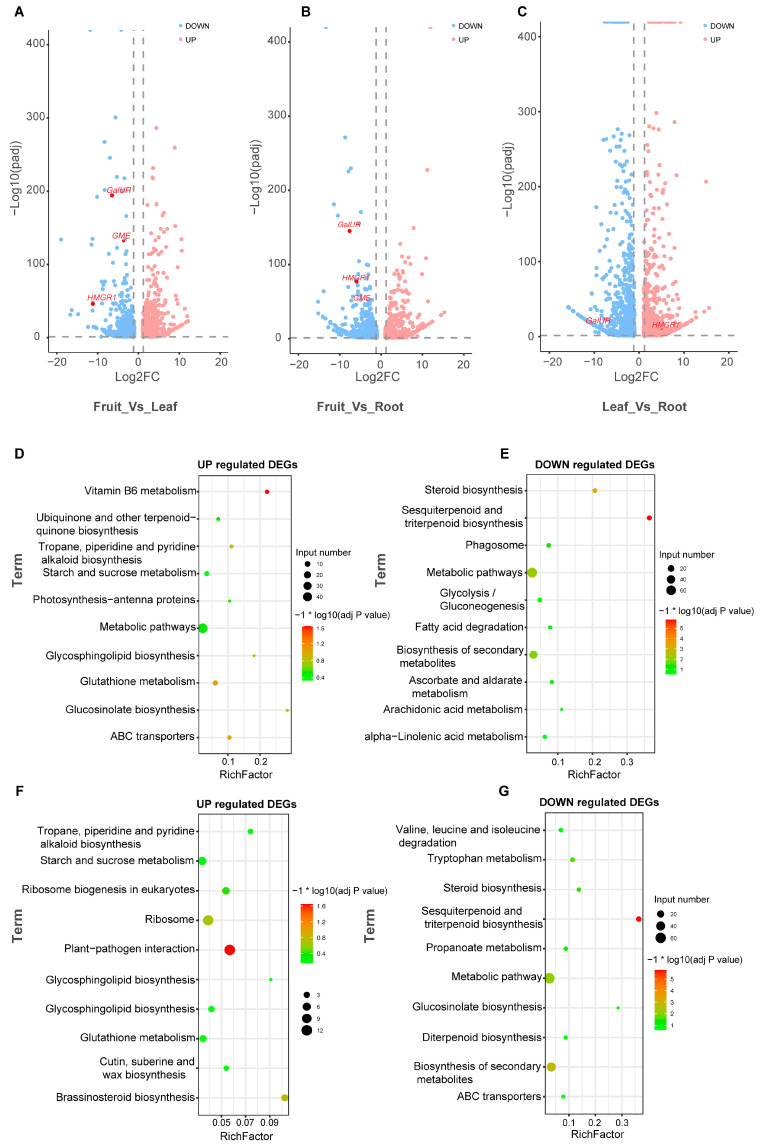
Volcano plots of differentially expressed genes (DEGs) and KEGG pathway enrichment analysis of DEGs in various organs of *R. roxburghii*. (**A**) Volcano plot of differentially expressed genes comparing fruit and leaves. (**B**) Volcano plot of differentially expressed genes comparing fruit and roots. (**C**) Volcano plot of differentially expressed genes comparing leaves and roots. The x-axis represents log2(FoldChange), and the y-axis represents the -log10 adjusted *p*-value (padj). Blue dots indicate down-regulated genes, while pink dots indicate up-regulated genes. Red dots denote genes involved in vitamin C metabolism. (**D**) KEGG pathway enrichment analysis of up-regulated differentially expressed genes comparing fruit and leaves. (**E**) KEGG pathway enrichment analysis of down-regulated differentially expressed genes comparing fruit and leaves. (**F**) KEGG pathway enrichment analysis of up-regulated differentially expressed genes comparing fruit and roots. (**G**) KEGG pathway enrichment analysis of down-regulated differentially expressed genes comparing fruit and roots.

Due to the higher content of triterpenoids in the fruit compared to other tissues, we thus compared the fruit and other organs separately to identify genes that may be involved in triterpenoid synthesis in the fruit. Compared to the fruit, the expression of *D*-galacturonate reductase (*GalUR*, Rr405094), GDP-*D*-mannose-3′, 5′-epimerase (*GME*, Rr600317), UDP-glucosyltransferase *UGT74B1* (Rr405006), and mevalonate hydroxymethyltransferase *HMGR1* (Rr101079) significantly decreased in the leaves and roots, while the expression of UDP-glucosyltransferase *UGT74B1* (Rr405005) increased significantly (Figure 4A,B). 

Given that *GalUR* and *GME* are mainly involved in the synthesis of ascorbic acid and vitamin C, this result suggests that the higher content of vitamin C in the fruit may be due to the up-regulation of genes involved in the vitamin C biosynthetic pathway, ultimately resulting in a higher accumulation of vitamin C in the fruit compared to the leaves and roots. Similarly, compared to the leaves, the expression of *GalUR* decreased in the roots, while the expression of *HMGR1* increased (Figure 4C). 

To gain further insights into the metabolic pathways associated with these DEGs, we selected the top 500 up-regulated and down-regulated genes from these DEGs. Using the KOBAS database (http://bioinfo.org/kobas/, accessed on 20 December 2020), we conducted KEGG pathway enrichment analysis separately for each comparison. The results indicate that among the DEGs compared between fruit and leaves, the up-regulated genes (genes with higher expression in leaves compared to fruit) mainly enriched with vitamin B6 metabolism (PYRIDOXIN (PYRODOXAMINE) 5′-PHOSPHATE OXIDASE [Rr504963], pyridoxal reductase 1 [Rr705141], PYRIDOXINE BIOSYNTHESIS 1.3 [Rr601261], PHOSPHOETHANOLAMINE/PHOSPHOCHOLINE PHOSPHATASE1 [Rr100758]), glutathione metabolism, and ABC transporter proteins. Conversely, the down-regulated genes (genes with lower expression in leaves compared to fruit) mainly enriched with sesquiterpenoid and triterpenoid biosynthesis squalene epoxidase 3 [*SE3*, Rr200341, Rr402844 and Rr402830], squalene epoxidase 2 [*SE2*, Rr200354 and Rr402843], β-amyrin synthase [β-*AS*, Rr105232] (Figure 4D,E and Table S3). Similarly, among the DEGs compared between fruit and roots, the up-regulated genes (genes with higher expression in roots compared to fruit) mainly enriched with plant–pathogen interactions. Conversely, the down-regulated genes (genes with lower expression in leaves compared to fruit) mainly enriched with sesquiterpenoid and triterpenoid biosynthesis (Figure 4F,G). Based on the comparison of DEGs across various tissues, it can be observed that the main metabolic pathways involved in the fruit primarily include the biosynthesis of sesquiterpenes, triterpenes, and secondary metabolites, while in the leaves, the main pathways involve the biosynthesis of sesquiterpenes and triterpenes. The metabolic pathways in the roots may mainly be related to ribosome metabolism and plant–pathogen interactions. 

### 2.4. CYP and UGT Are Potentially Involved in Kajiichigoside Synthesis in R. roxburghii

**Figure 5:**
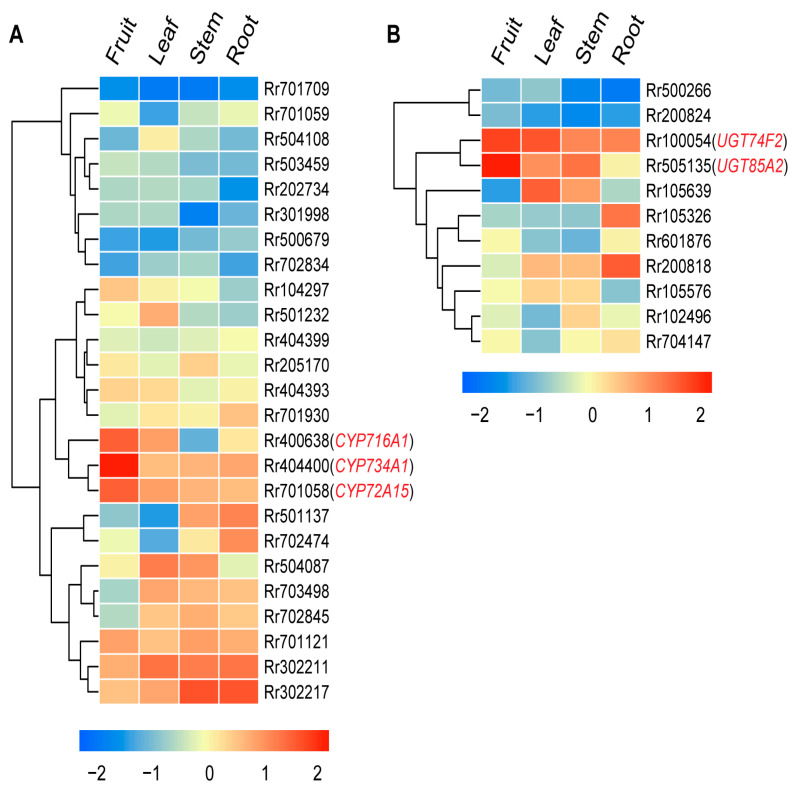
Expression patterns of *CYP* and *UGT* genes in *R. roxburghii*. (**A**) The expression patterns of cytochrome P450 family members (*CYP*) in fruit and leaves of *R. roxburghii*. (**B**) The expression patterns of *UGT* genes in fruit and leaves of *R. roxburghii*.

Given that both fruit and leaves can synthesize sesquiterpenes and triterpenes, which are likely closely related to the biosynthesis of kajiichigoside in *R. roxburghii*, we further analyzed the expression patterns of cytochrome P450 (CYP) family and glycosyltransferase (UGT) family genes in *R. roxburghii*. Compared to the leaves, cytochrome P450 family genes *CYP716A1*, *CYP734A1*, and *CYP72A15* are expressed at higher levels in the fruit (Figure 5 and Table S1). Similarly, glycosyltransferase genes *UGT74F2* and *UGT85A2* are expressed at higher levels in the fruit and lower levels in the leaves. We speculate that these highly expressed cytochrome P450 family genes and glycosyltransferase genes may be involved in the biosynthesis of kajiichigoside in *R. roxburghii* (Figure 5 and Table S1).

### 2.5. Expression Pattern with Cluster 12 Is Involved in Kajiichigoside Synthesis in R. roxburghii

**Figure 6:**
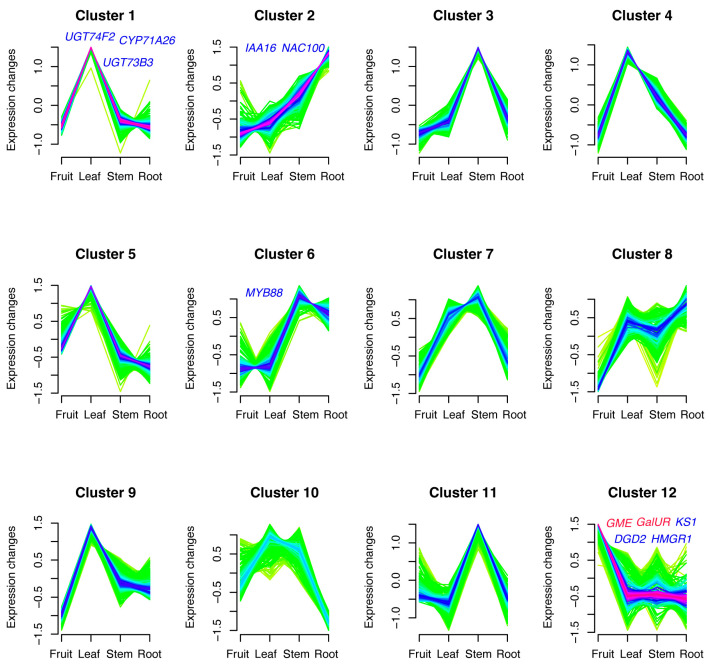
The gene expression patterns of the transcriptomes from various tissues (roots, stems, leaves, and fruit) of *R. roxburghii*.

Due to the higher content of kajiichigoside in the fruit compared to the leaves, we further explored the key genes involved in triterpenoid synthesis by utilizing time-series analysis (using the Mfuzz package, version 2.58.0) to categorize the expression patterns of *R. roxburghii* from different tissues (roots, stems, leaves, and fruit). We obtained 12 clusters that display distinct expression patterns (Figure 6). 

Cluster 1 represents an expression pattern characterized by high expression in the leaves gradually decreasing expression in the stems and roots, while Cluster 2 represents a pattern of gene expression gradually increasing in the fruit, leaves, stems, and roots. Cluster 3 exhibits the highest expression in the stems and the lowest expression in the roots and fruit, whereas Clusters 4 and 9 represent genes with the highest expression in the leaves and relatively lower expression in the fruit and roots. Similarly, Cluster 5 also shows relatively higher expression in the leaves and decreasing expression in the roots and stems. Clusters 6 and 7 display gene expression patterns with lower expression in the fruit but highest expression in the stems. Cluster 12 demonstrates the highest expression in fruit while gradually decreasing expression in the leaves, stems, and roots. Since the kajiichigoside content is highest in the fruit, we deduced that Cluster 12 may be the primary cluster related to kajiichigoside synthesis. Indeed, key genes involved in vitamin C synthesis (such as *GME* and *GalUR*) are present in this cluster. Furthermore, genes encoding squalene epoxidase 3 (*SQE3*, Rr200341), squalene epoxidase 2 (*SQE2*, Rr200354), KAURENE SYNTHASE (*KS1*, Rr301822), UDP-galactose-dependent digalactosyldiacylglycerol (DGDG) synthase (*DGD2*, Rr703028) and *bZIP29* (Rr201705) are present in the cluster 12 (Figure 6 and Table S2). This cluster likely represents a potential gene network involved in the kajiichigoside metabolism of fruit in *R. roxburghii*. 

Given that rosamultin glycoside preferentially accumulates in the stems (Figure 2), we also analyzed the cluster 7 as its expression patterns are consistent with the accumulation of rosamultin glycoside. *UGT75B1*(Rr704147), *CYP711A* (Rr205170), *CYP704A* (Rr702834), and *ACC2* (Rr505279) are present in this cluster (Figure 6 and Table S2). This cluster likely represents a potential gene network involved in the rosamultin glycoside metabolism of stem in *R. roxburghii*.

## 3. Discussion 

*R. roxburghii* is known to be rich in ascorbic acid, flavonoids, and triterpenoid compounds in its fruits, which typically function as antioxidants, anti-tumor, and anti-inflammatory agents, thereby offering significant potential for health benefits [1,2]. Triterpenoids, in particular, have garnered attention from chemists and biologists due to their medical and nutritional value. Consequently, understanding the molecular mechanisms governing the accumulation of triterpenoids could serve as a basis for designing or engineering *R. roxburghii* cultivars with modified metabolites. 

In this study, transcriptomic and metabolomic approaches were employed to investigate the accumulation patterns and regulatory mechanisms of triterpenoids in *R. roxburghii*. In other species, β-*AS*, *OSC* and *SE* have been shown to be correlated with the synthesis of pentacyclic triterpenoids [11,21,22]. Notably, we found that *SE2*, *SE3*, β-*AS*, and *OSC* (Rr105232 and Rr400924) were highly expressed in the fruits of *R. roxburghii* (Table S1). This finding is consistent with the significant accumulation of pentacyclic triterpenoids in *R. roxburghii* fruits (Figure 2). Based on these results, we propose that *SE2*, *SE3*, *OSC* and β-*AS* are likely involved in the biosynthesis of Euscaphic acid, Kaji-ichigoside F1 and rosamultin in *R. roxburghii*. Additionally, *SQE2*, *KS1*, *DGD2*, and *bZIP29* are likely associated with kajiichigoside metabolism in *R. roxburghii* fruit, while *UGT75B1*, *CYP711A*, *CYP704A*, and *ACC2* are likely contributors to the biosynthesis of rosamultin glycoside in *R. roxburghii* (Figure 6 and Table S2). 

The *CPY* and *UGT* families have been shown to play essential roles in shaping the structural diversity of triterpenoids across the plant kingdom [23,24,25]. Among the *CYPs*, *CYP716s* have been demonstrated to be a major contributor responsible for diversification of eudicot triterpenoid biosynthesis [23]. In contrast, it has been shown that members of the *UGT* family, including the *UGT71*, *UGT74*, *UGT85*, *UGT91*, and *UGT94* subfamilies, play a role in the glycosylation of plant pentacyclic triterpenoids [25,26,27]. In this study, we found that *CYP716A1*, *CYP734A1*, *CYP72A15*, *UGT74F2*, and *UGT85A2* had higher expression levels in fruits than in other organs in *R. roxburghii* (Figure 5), suggesting their likely involvement in the modification of pentacyclic triterpenoids. Taken together, our results have implications for the design and improvement of functional foods enriched with ascorbate and triterpenoids in *R. roxburghii*, although additional work is required to validate the function of candidate genes identified in this work.

The sensitive and selective detection of ursolic acid-type triterpenoids presents a significant challenge in high-performance liquid chromatography (HPLC) analysis. This limitation often hinders the simultaneous and accurate quantification of these valuable natural products within complex matrices like *R. roxburghii* extracts [28]. To address this, we present a novel HPLC method optimized for the targeted detection of multiple triterpenoid components. Therefore, our methods may help to quantify the content of triterpenoids in other plant species. 

## 4. Materials and Methods

### 4.1. Plant Material

The samples of *R. roxburghii* used in this study were obtained from the ‘Guinong 5’ variety cultivated at the nursery of the key laboratory of chemistry of natural products of the Chinese Academy of Sciences in Guizhou province, China. For the leaves, stems, and fruits of *R. roxburghii*, the samples were rapidly frozen with liquid nitrogen and stored at −80 °C after collection. Young leaves and old leaves, and young fruits and mature fruits were mixed in a 1:1 ratio before RNA isolation. For samples of the roots of *R. roxburghii*, we uprooted the entire plants, washed away the soil with sterile water, and placed the roots on ice. Upon returning to the laboratory, they were frozen at −80 °C for storage. All the samples were harvested in late July 2023. To ensure the consistency and comparability of the samples, all collected specimens were divided into two parts: one part was lyophilized at −5 °C for 72 h using a freeze-dryer (model not provided), followed by high-performance liquid chromatography (HPLC) analysis to determine the triterpenoid content in *R. roxburghii*; the other part was used for RNA-Seq analysis. Each sample contains three biological replicates.

### 4.2. HPLC Analysis and Triterpenoid Content Determination

#### 4.2.1. Extraction Process

The extraction process was adapted and optimized from Li’s method [29] to enhance the extraction efficiency of triterpenoid compounds from *R. roxburghii*. Initially, *R. roxburghii* root samples were dried in an oven at 65 °C to a constant weight, then ground and sieved through a No. 3 mesh to obtain a uniform powder. Precisely 2.00 g of the powdered sample was placed into a 250 mL conical flask, to which 50 mL of 70% ethanol was added as the extraction solvent. The conical flask containing the sample was then subjected to ultrasonic treatment for 0.5 h to increase the extraction efficiency, followed by transferring the sample to a round-bottom flask and heating under reflux for 2 h in a heating reflux apparatus to further extract triterpenoid compounds.

To ensure thorough extraction, the above extraction process was repeated three times consecutively. After each extraction, the extracts were combined to obtain a total triterpenoid extract from the *R. roxburghii* roots. The same method was applied to extract triterpenoids from the stems, leaves, and fruits of *R. roxburghii* to ensure the comprehensiveness and comparability of the experiment.

#### 4.2.2. Sample Preparation

(1) Standard Solution Preparation

Four types of *R. roxburghii* triterpenoid standards (self-made and its purity is determined to be ≥98% by HPLC detection), including kaji-ichigoside F1, rosamultin, euscaphic acid and 2α,19α-dihydroxyursolic acid were accurately weighed and placed into 10 mL volumetric flasks. Using 75% ethanol as the solvent, each standard was fully dissolved and brought up to the mark to prepare individual standard solutions. This procedure yielded standard concentrations as follows: kaji-ichigoside F1 1.003 mg/mL, rosamultin 1.014 mg/mL, euscaphic acid 1.802 mg/mL, and 2α,19α-dihydroxyursolic acid 1.998 mg/mL. These standard solutions were then used for subsequent high-performance liquid chromatography analysis to ensure the accuracy and reliability of the quantitative analysis. 

(2) Test Sample Solution Preparation

First, 2.00 g of powdered samples from the root, stem, leaf, and fruit parts of *R. roxburghii* were accurately weighed, and each placed into 250 mL conical flasks, with 50 mL of 70% ethanol added as the extraction solvent. The samples were treated in an ultrasonicator (SB25-12DTD ultrasonic instrument, Ningbo Xinzhi Biotechnology Co., Ltd., Ningbo, China) for 0.5 h to enhance the extraction efficiency (25 °C, 250 W), then transferred to round-bottom flasks and subjected to 2 h of heating under reflux. To ensure thorough extraction, this extraction step was repeated three times, with the liquids from each extraction combined.

The combined extracts were concentrated using a rotary evaporator to remove excess ethanol and then made up to 25 mL with 70% methanol in a volumetric flask. The solutions were filtered through a 0.45 µm membrane filter to prepare the test sample solutions. These solutions were then used for high-performance liquid chromatography (HPLC) analysis to quantitatively determine the content of the four triterpenoid compounds mentioned above in the *R. roxburghii* samples.

#### 4.2.3. Determination of Triterpenoid Content in Different Parts of *R. roxburghii*

(1) Sample Treatment

After establishing the optimal conditions for extracting four quality markers from *R. roxburghii*, these conditions were applied to samples from different parts of *R. roxburghii* (root, stem, leaves, and fruit) to extract and determine the content of triterpenoid markers. The total content percentage of the markers was calculated using the formula: Total Marker Content (%) = (Total Content of 4 Markers/Original *R. roxburghii* Material) × 100%. This step aims to accurately assess the distribution and content levels of markers in different parts of *R. roxburghii*, providing a scientific basis for further activity evaluation and application. 

(2) Instrument Parameters

An Agilent HPLC system was employed, utilizing a Thermo C18 column (250 mm × 4.6 mm, 5 µm particle size) for Agilent-1260 high-performance liquid chromatography analysis. The detection wavelength was set at 203 nm to achieve optimal signal detection sensitivity. The column temperature was maintained at 20 °C to ensure stability throughout the analysis. The flow rate was controlled at 1 mL/min, and the injection volume was 10 µL, ensuring adequate separation and detection efficiency of the samples.

The mobile phase consisted of acetonitrile (HPLC grade, Shanghai Aladdin Biochemical Technology Co., Ltd., Shanghai, China) (A) and a 0.05% trifluoroacetic acid (Tianjin Kemio Chemical Reagent Co., Ltd., Tianjin, China) aqueous solution (B). A gradient elution program was used for separation: from 0 to 15 min, mobile phase A increased from 25% to 30%; from 15 to 20 min, mobile phase A increased from 30% to 35%; from 20 to 60 min, a 35% A isocratic elution was performed. This gradient elution condition aimed to optimize the separation of each marker, ensuring high resolution and reproducibility of the detection results.

Each sample underwent three independent extractions, and error bars represent the standard deviation of the total triterpenoid content from the three repeats. Significant differences were determined by one-way ANOVA combined with Duncan’s multiple range test (*p* < 0.05).

#### 4.2.4. Methodological Investigation

Following the method described in [20], precision, stability, and repeatability tests were conducted on the *R. roxburghii* samples. The results showed that the relative standard deviations (RSD) were all less than 3%, demonstrating the stability and reliability of the method. This indicates that the HPLC analysis method adopted in this study possesses good precision and stability, capable of accurately determining the triterpenoid content in *R. roxburghii*, and provides reliable technical support for further compound analysis and functional studies. Through this series of stringent analytical conditions and methodological investigations, the accuracy and reproducibility of the experimental data are ensured, providing a solid foundation for research on triterpenoid compounds in *R. roxburghii*.

### 4.3. RNA Extraction and Library Construction

Total RNA was isolated from *R. roxburghii* leaf tissue using TRIzol^®^ reagent (Invitrogen, Carlsbad, CA, USA), following the manufacturer’s guidelines to ensure the extraction of high-quality RNA. The quality and purity of total RNA were assessed using the 2100 Bioanalyzer (Agilent Technologies, Santa Clara, CA, USA) and NanoDrop 2000 (Thermo Scientific, Wilmington, DE, USA). RNA-seq transcriptome libraries were prepared using the Illumina TruSeq™ RNA Sample Preparation Kit (San Diego, CA, USA) according to the manufacturer’s instructions. After library construction, sequencing was performed on the Illumina HiSeq X Ten platform with 2 × 150 bp paired-end sequencing, with three biological replicates per sample to ensure data reliability and repeatability.

### 4.4. Transcriptome Data Processing and Alignment

Raw reads were cleaned by removing adapters and low-quality sequences using Fastp software (Version 0.23.4) [30]. Subsequently, the cleaned reads were mapped to the assembled genome sequences of *R. roxburghii* [31] using STAR software (Version STAR_2.5.2b) [32]. Following mapping, the reads were counted, and their abundance was quantified using RSEM (Version 1.3.3) to obtain TPM and the expected count values [33].

### 4.5. Differential Expression Analysis and Functional Annotation

The differential expression among samples was identified using the DESeq2 package (Version 1.30.0) [34]. Differentially expressed genes (DEGs) were identified based on an absolute value of the Log2 (FoldChange)  ≥  1 and *p* < 0.05. Functional annotation of DEGs and KEGG functional enrichment analyses were conducted using the eggNOG 5.0 and KOBAS database [35,36]. The expressed gene after log2 transformation were classified into 12 clusters by Mfuzz based on their expression value [37].

## 5. Conclusions

In this study, we conducted HPLC profiling of four triterpenoids from the roots, stems, leaves, and fruits of ‘Guinong No. 5’ *R. roxburghii*, and performed transcriptomic analysis of the roots, stems, leaves, and fruits of this variety to analyze the distributions of triterpenoid compounds among different plant parts and unveil a potential gene network implicated in the biosynthesis of both ascorbate and triterpenoid compounds in this variety. The HPLC profiling results showed that different triterpenoids exhibit specific accumulation patterns in *R. roxburghii*. Among them, kaji-ichigoside F1 accumulates mostly in the fruits and roots, while rosamultin accumulates mostly in the stems and fruits. The transcriptomic analysis revealed that genes involved in vitamin C, sesquiterpenoid, and triterpenoid biosynthesis were significantly up-regulated in the fruits of *R. roxburghii*, likely contributing to higher contents of these compounds in *R. roxburghii* fruits. Additionally, preferential expression of different *CYPs* and *UGTs* genes, such as *CYP716A1*, *CYP734A1*, *CYP72A15*, *UGT74F2*, and *UGT85A2*, in different tissues of *R. roxburghii* is likely responsible for the synthesis of Kajiichigoside in *R. roxburghii* fruits. 

## Figures and Tables

**Table 1:** Triterpene contents in *R. roxburghii* samples of different tissues by HPLC.

Tissue	Kaji-Ichigoside F1	Rosamultin	Euscaphic Acid	2α,19α-Dihydroxyursolic Acid
Fruit	1.29 ± 0.02	0.14 ± 0.01	0.62 ± 0.02	0.72 ± 0.01
Stem	0.34 ± 0.01	0.48 ± 0.02	0.22 ± 0.02	0.36 ± 0.03
Root	0.76 ± 0.03	0.02 ± 0.01	0.38 ± 0.03	0.33 ± 0.02
Leaf	0.51 ± 0.03	0.03 ± 0.01	0.27 ± 0.01	0.31 ± 0.01

## Data Availability

Transcriptome sequencing data were deposited in the CNSA database (https://db.cngb.org) under project number CNP0005667.

## Supplementary Materials

The supporting information can be downloaded at: https://www.mdpi.com/article/10.3390/ijms25105517/s1.
